# Precision Medicine in Temporomandibular Joint Disorders: A Synovial Fluid Biomarker-Based Literature Review

**DOI:** 10.3390/medicina62061179

**Published:** 2026-06-17

**Authors:** Francesco Maffìa, Francisco Salvado, Paola Bonavolontà, Henrique José Cardoso, David Sanz, Stefania Troise, Gianluca Renato De Fazio, Giovanni Dell’Aversana Orabona, David Faustino Ângelo

**Affiliations:** 1 Instituto Português da Face, 1500-493 Lisbon, Portugal; henrique.cardoso@ipface.pt (H.J.C.); david.sanz@ipface.pt (D.S.); david.angelo@ipface.pt (D.F.Â.); 2 Faculty of Medicine, University of Lisbon, 1649-028 Lisbon, Portugal; 3 Clinica Universitária de Estomatologia, Centro Hospitalar Universitário Lisboa Norte (CHUNL), 1349-019 Lisbon, Portugal; fjsalvado2002@yahoo.com; 4 Maxillofacial Surgery Unit, Department of Neurosciences, Reproductive and Odontostomatological Sciences, University of Naples “Federico II”, 80131 Naples, Italy; paola.bonavolonta@unina.it (P.B.); gianludefazio@gmail.com (G.R.D.F.); giovanni.dellaversanaorabona@unina.it (G.D.O.); 5 Serviço de Estomatologia, Hospital de Egas Moniz, Centro Hospitalar de Lisboa Ocidental, 1349-019 Lisbon, Portugal; 6 Centre for Rapid and Sustainable Product Development, Polytechnic Institute of Leiria, 2430-028 Leiria, Portugal

**Keywords:** temporomandibular joint, temporomandibular disorders, synovial fluid, biomarkers, proteomics analysis

## Abstract

*Background and Objectives:* Temporomandibular disorders (TMDs) encompass a broad spectrum of functional and structural abnormalities of the temporomandibular joint (TMJ). Conventional diagnostic tools, although essential, often fail to capture the underlying biochemical mechanisms driving disease progression. Synovial fluid (SF), by virtue of its direct proximity to intra-articular tissues, represents an accessible biological matrix for identifying molecular signatures of inflammation, cartilage degradation, lubrication failure, oxidative stress, and angiogenic activation. The objective of this review is to synthesize current evidence on SF proteomics in TMD and evaluate its potential translational value in precision medicine. *Materials and Methods:* A narrative review of the literature was conducted on PubMed to identify human studies focused on SF proteomic and biochemical biomarkers in TMD. Eligible studies included original research articles assessing SF composition in relation to specific TMJ pathologies, diagnostic categories, or clinical phenotypes. Extracted data included study design, sample characteristics, analytic methodology, biomarkers investigated, and key findings. Google Gemini (Google LLC, Mountain View, CA, USA) was used as an AI-assisted tool to support language editing and manuscript writing during the preparation of this article. The use of this tool was limited to linguistic refinement; all scientific content, data interpretation, and conclusions were formulated and verified by the authors. *Results:* Across the analyzed studies, TMD phenotypes—particularly disc displacement with or without reduction (DDwR, DDwoR) and osteoarthritis (OA)—were characterized by consistent alterations in cytokines (IL-1β, IL-6, IL-8, TNF-α), extracellular matrix (ECM) components (aggrecan, glycosaminoglycans (GAGs), decorin, MMP-2, MMP-9), lubrication molecules (lubricin/PRG4), oxidative stress mediators (myeloperoxidase (MPO), nitric oxide (NO), glutathione peroxidase (GPX)), adipokines (chemerin, resistin, adiponectin), and angiogenic factors (vascular endothelial growth factor (VEGF), fibroblast growth factor-2 (FGF-2)). Recent liquid chromatography–tandem mass spectrometry (LC–MS/MS) analyses further revealed phenotype-specific protein clusters and pathways related to inflammation, ferroptosis, hypoxia signaling, and proteoglycan metabolism. *Conclusions:* Current evidence suggests that SF proteomics and multi-analyte biomarker profiling offer a promising, hypothesis-generating approach for understanding the biological mechanisms underlying TMD. The integration of proteomic, metabolic, and inflammatory markers holds future potential for diagnostic panel development; however, prospective clinical validation is still required before SF-based molecular profiling can be implemented as a precision medicine tool in TMJ disorders.

## 1. Introduction

Temporomandibular disorders (TMDs) are defined as musculoskeletal disorders affecting the temporomandibular joint (TMJ), the masticatory muscles, or both, as well as surrounding structures [1]. TMDs include a wide-ranging spectrum of conditions, including locoregional pain, restricted jaw movement with mouth opening limitation, and joint sounds, like clicking [2]. The diagnosis of TMJ disorders is complex, and influenced by anatomical, functional, radiological factors [3]. Conventional diagnostic methods for TMJ disorders, such as clinical evaluation and imaging techniques, often fail to capture the underlying biochemical complexity of these conditions [4]. Extensive research on the etiology and pathophysiology of TMJ disorders at the cellular and molecular levels has underscored the critical role of the synovial membrane and synovial fluid (SF) in their pathogenesis [5]. Consequently, there is increasing emphasis on the biochemical analysis of changes in SF composition [6].

SF primarily serves as a biological lubricant for joints, minimizing friction between the articular cartilage surfaces [7]. Additionally, it acts as a reservoir of nutrients for adjacent tissues and facilitates the transport of cytokines [8]. Its proximity to tissues affected by orthopedic conditions, such as osteoarthritis (OA), rheumatoid arthritis (RA), juvenile arthritis, spondyloarthritis, osteochondrosis, and synovial sepsis, makes SF a valuable medium for studying these pathologies [9]. The minimally invasive nature of its collection through joint puncture further enhances its utility for exploring disease molecular mechanisms and identifying potential biomarkers [10].

Cytokines, proteins, and growth factors in SF play key roles in regulating joint immune and metabolic functions [11]. Produced by synovial cells, chondrocytes, or derived from plasma, these cytokines are classified as proinflammatory (e.g., IL-1b, IL-2, IL-6, TNF) or anti-inflammatory (e.g., IL-10, IFN-γ) [11]. In inflammatory joint conditions or injuries, proinflammatory cytokines dominate, disrupting the balance and contributing to cartilage and bone degeneration [12]. In TMDs, elevated cytokines like IL-1b, TNF, and IL-6 are linked to conditions such as OA, internal derangement, and closed lock [12]. These inflammatory mediators are critical for understanding TMJ pathology, as they drive pain, tissue degradation, and potential growth impairments, particularly in younger individuals [13].

Proteomics analysis has extended these analytical capabilities to allow a comprehensive study of the biological processes in the SF of the TMJ [14]. Proteomic methods, like mass spectrometry, allow for the identification and quantification of proteins in SF, as well as the study of protein–protein interactions [15]. Unlike cytokine analysis, proteomics provides a broader, more comprehensive view of protein changes and interactions [15]. Understanding the proteins involved in TMDs may reveal those responsible for persistent pain, disk degradation, clicking sounds, or joint destruction [16]. Currently, several key proteins have been found in SF of TMD patients like proinflammatory cytokines (IL-1β, TNF-α, IL-6), matrix metalloproteinases, tissue inhibitors of metalloproteinases, biomarkers of cartilage degradation such as aggrecan and glycosaminoglycans [17]. Comparing the proteomic profiles of TMD SF to normal controls can help identify disease-specific proteins and potential biomarkers [14]. This narrative review aimed to evaluate the clinical potential of temporomandibular joint synovial fluid analysis, summarizing known biomarker classes and comparing analytical techniques applied across the reviewed literature.

## 2. Materials and Methods

The initial phase involved a narrative literature review utilizing the PubMed database to identify a set of potentially relevant studies examining the role of SF proteome analysis in TMJ disorders. The PubMed database was selected as the primary source due to its comprehensive coverage of biomedical and dental literature, including all major journals relevant to TMJ research and synovial fluid analysis. The search strategy included the following terms: ((“Synovial Fluid”[MeSH] OR “synovial fluid proteomics”) AND (“Temporomandibular Joint”[MeSH] OR “TMJ” OR “Temporomandibular Disorders”[MeSH]) AND (“Proteomics”[MeSH] OR “protein analysis” OR “biomarkers”)). The second phase involved refining the literature review to focus on studies related to the SF proteomic analysis in TMJ pathology. The inclusion criteria were: (1) human studies; (2) original research articles examining synovial fluid composition, biochemical markers, or proteomic/metabolomic profiles in relation to TMDs; (3) articles published in English; (4) studies providing data on at least one identifiable biomarker class in TMJ SF. Exclusion criteria comprised: animal studies, conference abstracts, editorials, case reports with fewer than three patients, studies exclusively examining non-SF biological matrices (serum or saliva only), and articles not providing original data. The literature search was performed up to March 2025 and was not registered in a prospective review registry. As this is a narrative review, no formal quality assessment or risk-of-bias tool was applied, and the findings should be interpreted accordingly. For each selected paper, the following data were extracted: first author, publication year, study population, type of pathology, biological material analyzed, collection technique, analytical method, main biomarkers investigated, and key findings.

## 3. Results

A total of 24 studies met the inclusion criteria and were included in this review. Their main characteristics—including study design, patient population, sample size, analytical method, principal biomarkers investigated, and key findings—are summarized in Table 1.

### 3.1. Subjects and TMJ Pathologies

Across the reviewed studies, the majority of subjects presented with TMD characterized by disc displacement with/without reduction (DDwR/DDwoR) associated or not to OA of the TMJ [21]. These pathological entities were the focus of most investigations, with a smaller number of studies enrolling healthy controls for baseline comparisons [22]. DDwoR was also studied in association to presence/absence of condylar resorption and to presence/absence of bone formation [21]. In some studies, Wilkes classification was used to stratify patients to be included, in particular, stage III-IV-V were specifically examined [18].

### 3.2. TMJ Analyzed Material

Synovial fluid was the main material analyzed in most of the reviewed studies [19]. Its easy access through minimally invasive arthrocentesis and direct contact with intra-articular metabolic processes made it the preferred sample [4]. TMJ SF can also be collected during arthroscopy when dilating the TMJ capsule or during open surgery by directly collecting just before capsular incision [20]. Although less common, some studies focused on complex surgical cases, such as those requiring functional condylectomy, where tissue analysis is crucial [33]. An unusual case was noted in a study that analyzed and isolated proteins from fibroblasts taken from OA-affected TMJ synovium, diverging from the typical fluid-based methods [25]. In several studies included in this review, additional biological materials beyond SF were analyzed, including synovial membrane, retrodiscal tissue, and disc specimens obtained during open TMJ surgery or arthroscopy [30]. These samples were processed to add to the SF data and were specifically used in proteomic workflows or histological–molecular correlation studies [14]. The Excel extraction sheet confirmed that about one-fourth of the included studies used a dual approach—analyzing both fluid and tissue—to strengthen links between intra-articular biochemical markers and structural or degenerative changes [30].

### 3.3. Technique of Collection

The collection of TMJ SF predominantly employed arthrocentesis, typically involving the injection and aspiration of sterile saline solution [26]. Several variations in dilution amount or aspiration maneuvers were observed. The average volume of collecting solution reported across studies is 2 mL, that may be injected or reinjected several times [28]. Injection volumes ranged from 1 to 4 mL across the included studies. Fluid collection is achieved either by capsule elastic retraction following distension, or by direct intra-articular aspiration using a push-and-pull technique [26]. Several studies modified the standard arthrocentesis technique by incorporating additives to optimize sample retrieval, adopting in some cases the Alstergren method [23]. This method consists of creating a washing solution adding a mix made of 22% of hydroxocobalamin (Behepan 1 mg) to 9 mL of saline. Hydroxocobalamin may be used as external marker that allows quantification of total SF and calculation of the dilution factor via spectrophotometry, enabling accurate measurement of true joint fluid volume with a detection limit below 1% [23]. While arthrocentesis was the standard, other collection methods included surgical approaches for direct synovial tissue harvesting during open joint procedures [20,33]. More recently, a closed-loop arthrocentesis technique has been introduced, employing a dual-needle sealed circuit to minimize fluid dilution and loss, thereby improving total protein yield and sample quality for downstream biochemical analyses [32].

### 3.4. Type of Analytic Test

In terms of analytic methodologies, enzyme-linked immunosorbent assay (ELISA) was the most commonly used technique for quantifying cytokines, matrix enzymes, and other low-abundance biomarkers in SF [34]. Spectrophotometric and multiplex platforms such as Luminex were also employed to evaluate multiple inflammatory mediators simultaneously [27]. Molecular analyses including PCR and Western blotting were primarily reserved for tissue-derived samples. More advanced studies adopted mass spectrometry-based proteomic workflows—mainly LC–MS/MS and related approaches—to identify broader protein profiles and pathway-level signatures associated with inflammation, degeneration, oxidative stress, and metabolic alterations [14]. A smaller number of studies incorporated untargeted metabolomics or combined tissue–fluid analyses, reflecting a gradual shift towards multi-omics characterisation of TMD [31].

### 3.5. Main Biomarkers

The main biomarkers investigated across the included studies were predominantly related to inflammation, extracellular matrix (ECM) degradation, oxidative stress, lubrication, angiogenesis, and metabolic regulation. Proinflammatory cytokines—IL-1, IL-1β, IL-6, IL-8, IL-10, IL-11, TNF-α, and IFN-γ—were the most commonly assessed markers [11,12]. Oxidative stress biomarkers included myeloperoxidase (MPO), glutathione peroxidase (GPX), and nitric oxide (NO) [24]. ECM-related biomarkers comprised aggrecan, glycosaminoglycans (GAGs), decorin, MMP-2, and MMP-9, reflecting fibrocartilage degradation [29].

## 4. Discussion

### 4.1. Temporomandibular Joint Synovial Fluid Characteristics

Synovial fluid is a viscous, non-Newtonian fluid found in the cavities of synovial joints. Its principal role is to reduce friction between the articular cartilage of synovial joints during movement [7]. Synovial fluid is composed primarily of hyaluronic acid, lubricin, and interstitial fluid filtered from blood plasma [7,8]. The SF within the TMJ exhibits distinct physiological characteristics compared to the SF of other musculoskeletal joints [19]. Relative to other joint spaces, the TMJ SF has a lower volume but higher concentrations of proteins, glycoproteins, lipids, and cellular constituents [6,23]. This unique composition reflects the specialized anatomical and functional features of the TMJ, where the SF serves as a crucial lubricating medium that facilitates the proper biomechanical functioning of the articular surfaces [6]. Key physiological functions of SF include:

• *Lubrication*: Synovial fluid provides both boundary and fluid-film lubrication to the articular cartilage, minimizing friction and wear during joint movement [6,7]. Hyaluronic acid and lubricin are key components contributing to these lubrication mechanisms.
• *Nutrient and waste transport*: As cartilage is avascular, SF plays a crucial role in delivering nutrients to and removing metabolic waste products from the chondrocytes within the cartilage matrix [8].
• *Shock absorption:* The viscoelastic properties of SF contribute to shock absorption within the joint, protecting the articular cartilage and underlying bone from impact forces [6].
• *Homeostasis:* SF helps maintain a stable joint environment by regulating temperature, pH, and electrolyte balance. It also contains phagocytic cells that remove debris and microbes [6].

In OA and other joint diseases, the composition and properties of SF can be altered, contributing to pain, inflammation, and impaired joint function [14]. For instance, the concentration and molecular weight of hyaluronic acid are often reduced in OA, affecting the fluid’s lubricating and viscoelastic properties. Analysis of SF can provide valuable diagnostic information about joint health and disease [4,10]. The normal TMJ SF proteome comprises a balanced and tightly regulated mixture of molecular components, including cytokines, chemokines, enzymes, and growth factors, which are involved in the physiological processes governing joint homeostasis [5,12]. These biomolecules play pivotal roles in regulating joint immune responses, maintaining the structural integrity of articular cartilage, and facilitating nutrient transport to surrounding tissues. The precise composition and interactions of these SF constituents are essential for preserving the normal function and health of the TMJ.

### 4.2. Synovial Fluid Physiopathology

The SF plays a pivotal role in the pathogenesis of TMD. The normal SF proteome, which encompasses the molecular components involved in the physiological functioning of the TMJ, has been consistently reported to undergo significant alterations in various TMDs [13]. These changes are attributed to the complex inflammatory processes and tissue damage associated with TMDs, leading to the release of numerous proteins, enzymes, and molecular mediators into the SF [14]. Compared to healthy controls, the SF of patients with TMDs exhibits elevated levels of proinflammatory cytokines, such as IL-1β, TNF-α, and IL-6, as well as matrix metalloproteinases and their tissue inhibitors [11,15]. These inflammatory mediators contribute to the progression of TMDs by promoting pain, joint stiffness, and structural alterations in the joint [16]. Furthermore, the SF of TMD patients may also display increased levels of biomarkers associated with cartilage degradation, including aggrecan, glycosaminoglycans, and other ECM components [22]. The presence of these proteins in the SF reflects the ongoing pathological processes affecting the articular cartilage and other joint tissues, underscoring the dynamic and complex nature of the joint’s pathophysiology. The alterations in the SF proteome can provide valuable insights into the underlying mechanisms contributing to the development and progression of TMDs, potentially aiding in the identification of novel diagnostic and therapeutic targets. It is important to acknowledge, however, that multiple clinical confounding variables may substantially influence the SF biomarker profile. Patient age and biological sex are established modulators of joint inflammation and cytokine secretion. Systemic inflammatory conditions—particularly rheumatoid arthritis—share overlapping molecular signatures with TMD and may confound biomarker specificity. Current pharmacological management, including non-steroidal anti-inflammatory drugs (NSAIDs), corticosteroids, and prior intra-articular injections of hyaluronic acid, can transiently suppress cytokine levels and alter the proteomic milieu of the SF [28]. Metabolic conditions such as obesity and insulin resistance influence adipokine expression, while smoking status affects oxidative stress markers. Few of the reviewed studies systematically controlled for these variables, which limits the comparability and generalizability of reported findings. The biomarker expression profiles across TMD phenotypes and healthy controls are summarised in Table 2.

**Table 2:** Biomarker expression profiles across TMD phenotypes and healthy controls. Levels are reported relative to healthy controls based on evidence from reviewed studies. ↑ mildly elevated; ↑↑ moderately elevated; ↑↑↑ markedly elevated; ↓ mildly reduced; ↓↓ moderately reduced; ↓↓↓ markedly reduced; ↔ no significant change; NR: not reported. DDwR: disc displacement with reduction; DDwoR: disc displacement without reduction; OA: osteoarthritis; IL: interleukin; TNF-α: tumour necrosis factor-alpha; IFN-γ: interferon-gamma; MMP: matrix metalloproteinase; GAG: glycosaminoglycan; PRG4: proteoglycan 4 (lubricin); PGE2: prostaglandin E2; MPO: myeloperoxidase; NO: nitric oxide; GPX: glutathione peroxidase; VEGF: vascular endothelial growth factor; FGF-2: fibroblast growth factor-2.

Biomarker/Class	Healthy Controls	DDwR	DDwoR	OA
***Inflammatory cytokines***				
IL-1beta	Baseline/Low	↑	↑↑	↑↑↑
IL-6	Baseline/Low	↑	↑↑	↑↑↑
IL-8	Baseline/Low	↑	↑↑	↑↑
TNF-α	Baseline/Low	↑	↑↑	↑↑
IFN-γ	Baseline/Low	↑	↑	↑
IL-10 (anti-inflammatory)	Detectable	↔	↑	↑
***ECM degradation***				
MMP-2	Low	↑	↑↑	↑↑
MMP-9	Low/Absent	↑	↑↑	↑↑↑
MMP-7	Low	NR	↑	↑↑
Aggrecan/GAG fragments	Baseline	↑	↑↑	↑↑↑
Decorin	Baseline	↑	↑↑	↑↑
***Lubrication***				
Lubricin/PRG4	High	↓	↓↓	↓↓↓
Hyaluronic acid	High	↓	↓↓	↓↓
PGE2	Low	↑	↑↑	↑↑
***Oxidative stress***				
Myeloperoxidase (MPO)	Low	↑	↑↑	↑↑
Nitric oxide (NO)	Low/Absent	↑	↑↑	↑↑↑
GPX	Present	↑	↑	↑↑
***Angiogenesis***				
VEGF	Low	↑	↑↑	↑↑
FGF-2	Low	↑	↑↑	↑↑
***Adipokines & metabolic mediators***				
Leptin	Low	↑	↑	↑↑
Chemerin	Low	↑	↑↑	↑↑
Adiponectin/Resistin	Detectable	NC/NR	↑	↑

### 4.3. Biomarker–Phenotype Relationships in Temporomandibular Disorders

Recent studies analyzing human TMJ SF have demonstrated that specific molecular patterns correspond to distinct inflammatory and degenerative TMD phenotypes. Rather than functioning as isolated findings, these biomarkers reflect interconnected biological pathways—encompassing synovial inflammation, extracellular matrix breakdown, impaired lubrication, oxidative stress, and angiogenic activation—that collectively characterize the clinical and structural progression of TMD [16].

#### 4.3.1. Inflammatory Cytokines

Proinflammatory cytokines are the most extensively studied molecular group in TMJ SF. Elevated concentrations of IL-1β, IL-6, IL-8, IL-11, TNF-α, and IFN-γ have been documented in internal derangement and DDwoR, with levels typically decreasing after arthrocentesis or intra-articular therapy [26]. Their importance is reinforced by systematic reviews demonstrating that IL-6, TNF-α, and IL-1β are reproducibly increased in TMJ OA and closed lock, irrespective of sampling or analytical technique [11]. Cytokines, therefore, serve as robust indicators of active synovitis and play a central role in pain generation, chemotaxis, and joint effusion. However, conflicting evidence exists regarding the magnitude and consistency of these elevations. Some studies failed to detect statistically significant differences in IL-1β or TNF-α concentrations between DDwR and healthy controls, possibly reflecting the early and partially reversible nature of disc displacement with reduction [5,27]. Methodological variability—including differences in ELISA sensitivity, SF dilution volumes, and patient selection—may account for a substantial proportion of the reported inter-study discrepancies.

#### 4.3.2. Matrix and Cartilage Degradation Biomarkers

TMJ degeneration is strongly linked to enzymatic and structural biomarkers of ECM breakdown. Increased expression of MMP-2 and MMP-9, both in SF and synovial membrane, has been shown in degenerative joints, confirming their role in collagenolysis and fibrocartilage remodeling [29]. Structural ECM components, particularly aggrecan fragments, glycosaminoglycans, and decorin, are elevated in joints with localized pain and radiographic degeneration [22]. These molecules reflect the biomechanical deterioration of the disc–condyle complex and may help distinguish acute inflammatory TMD from chronic degenerative disease. Nevertheless, the specificity of ECM markers for TMD remains uncertain, as similar MMP elevations have been reported in rheumatoid arthritis and knee osteoarthritis, complicating their use as TMD-specific diagnostic targets. Furthermore, studies vary considerably in which MMP isoforms are assessed and the analytical thresholds applied, limiting cross-study comparability.

#### 4.3.3. Lubrication Molecules

The TMJ relies heavily on boundary lubrication due to its fibrocartilaginous surfaces and complex kinematics. Lubricin (PRG4) is a critical molecule in this system, and its concentration decreases progressively with advancing Wilkes stage, suggesting that loss of lubrication contributes to symptom persistence and structural deterioration [7]. Lubricin reduction may also potentiate friction-induced inflammation, forming a self-amplifying degenerative loop. Despite this consistent directional trend, the precise threshold of lubricin reduction associated with symptom onset has not been established, and the degree of reduction relative to healthy controls varies considerably across studies depending on collection technique and dilution correction methodology.

#### 4.3.4. Oxidative Stress Biomarkers

Oxidative stress contributes significantly to TMD pathogenesis. Studies have demonstrated increased levels of myeloperoxidase (MPO), nitric oxide (NO), and glutathione peroxidase (GPX) in symptomatic joints, with MPO showing a notable reduction following hyaluronic acid therapy [28]. These biomarkers reflect intracellular stress responses and may offer value in monitoring therapeutic efficacy. Conflicting data exist, however, regarding GPX behaviour: some studies report elevated GPX as a compensatory antioxidant response, while others find no significant difference from controls, suggesting that the balance between oxidative challenge and antioxidant capacity may vary with disease stage and individual patient factors.

#### 4.3.5. Angiogenic Factors

Angiogenesis plays a key role in synovitis and joint remodeling. Elevated synovial levels of vascular endothelial growth factor (VEGF) and fibroblast growth factor-2 (FGF-2) have been reported in patients with TMJ internal derangement and OA, with both factors decreasing after intra-articular administration of therapeutics [10]. Their expression suggests ongoing vascular proliferation and synovial irritation, aligning TMJ degeneration with mechanisms observed in larger arthritic joints. The specificity of VEGF and FGF-2 for TMD pathology, however, remains uncertain, as these factors are broadly upregulated in any inflamed synovial tissue. No study to date has demonstrated that angiogenic SF markers can discriminate TMDs from other inflammatory arthropathies, and their predictive value for treatment response has not been evaluated.

#### 4.3.6. Adipokines

Adipokines have emerged as important modulators of TMJ inflammation. Chemerin, adiponectin, resistin, and apelin show significant alterations in TMJ SF, correlating with pain intensity, localized joint inflammation, and disc displacement [31]. Chemerin, in particular, shows unexpectedly high TMJ synovial concentrations, suggesting a unique metabolic–inflammatory interplay within TMJ tissues. The direction and magnitude of adiponectin changes, however, remain inconsistent across studies: while some report elevated adiponectin in inflammatory TMD, others find no significant deviation from controls or even reduced concentrations in advanced OA, reflecting the complex and context-dependent roles of this adipokine in joint tissues.

#### 4.3.7. Tissue–Fluid Discordance

**Figure 1:**
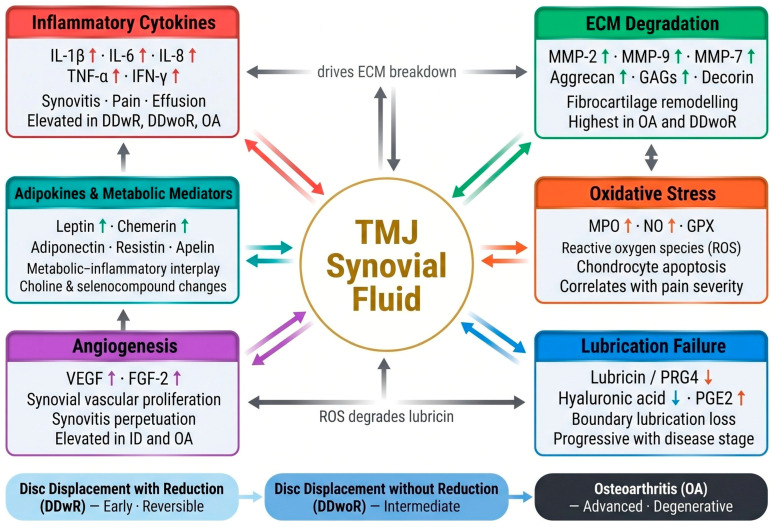
Molecular pathways and biomarker classes identified in temporomandibular joint (TMJ) synovial fluid across TMD phenotypes. Bidirectional arrows between the central TMJ synovial fluid node and each pathway cluster represent the reciprocal exchange of molecular mediators. Grey directional arrows indicate key inter-pathway crosstalk. ↑ elevated; ↓ reduced compared to healthy controls or early-stage disease. DDwR: disc displacement with reduction; DDwoR: disc displacement without reduction; OA: osteoarthritis; ECM: extracellular matrix; GAGs: glycosaminoglycans; MPO: myeloperoxidase; NO: nitric oxide; GPX: glutathione peroxidase; PRG4: proteoglycan 4 (lubricin); PGE2: prostaglandin E2; VEGF: vascular endothelial growth factor; FGF-2: fibroblast growth factor-2; IL: interleukin; TNF-α: tumour necrosis factor-alpha; IFN-γ: interferon-gamma; MMP: matrix metalloproteinase; ID: internal derangement.

A critical observation comes from studies comparing synovial tissue and SF collected simultaneously during surgery. Except for IL-8, cytokine concentrations showed poor correlation between the two matrices, indicating that SF alone may not fully capture intra-articular inflammatory activity [12]. This underscores the importance of paired tissue–fluid studies and suggests that some molecular events remain compartmentalized within the synovial membrane. Taken together, the reviewed studies display substantial methodological heterogeneity: sample sizes range from fewer than 10 to over 100 joints, SF collection volumes vary from 1 to 4 mL, dilution correction is applied inconsistently, and analytic platforms span ELISA, multiplex arrays, and mass spectrometry. This heterogeneity precludes meta-analytic synthesis and renders direct quantitative comparisons unreliable. Standardization of collection and analytical protocols is therefore a prerequisite for generating reproducible, clinically actionable biomarker data in TMD research. The key molecular pathways and inter-pathway crosstalk are illustrated in Figure 1.

### 4.4. Recent Advances in TMJ Synovial Fluid “Omics” for Temporomandibular Disorders

#### 4.4.1. Proteomics of TMJ Synovial Fluid

High-resolution proteomic analyses have refined the molecular stratification of TMD, revealing protein clusters that differentiate mechanical, inflammatory, and degenerative phenotypes beyond what can be achieved with classical biomarker profiling. In the nanoLC–MS/MS study by Doetzer et al., SF samples from patients with DDwoR, condylar hyperplasia, and mandibular dislocation demonstrated distinct proteomic signatures, including molecules involved in oxidative stress regulation, ECM turnover, metabolic adaptation, and cell–matrix communication [14]. Similar proteomic pathway clusters—particularly those related to ECM remodeling and oxidative stress—have been described in broader joint proteomics analyses [15].

Key proteins included Heat Shock Protein Beta-1 (HSPB1), Serpin Family F Member 1—Pigment Epithelium-Derived Factor (SERPINF1), Thrombospondin-3 (THBS3), Tenascin-C (TNC), Galectin-1 (LGALS1), Lumican (LUM), Pyruvate Kinase M1/2 (PKM), S100 Calcium-Binding Protein A10 (S100A10), Alpha-1 Antitrypsin (SERPINA1), Extracellular Superoxide Dismutase (SOD3), Transferrin (TF), and Transforming Growth Factor Beta-Induced Protein (TGFBI). These proteins form interconnected modules involved in cytoskeletal regulation, antioxidant defense, fibrocartilage remodeling, and inflammation–mechanical coupling.

Analysis of disc tissue further enriched the proteomic landscape, revealing Peroxiredoxin-1 (PRDX1), Peroxiredoxin-2 (PRDX2), Proline/Arginine-Rich End Leucine-Rich Repeat Protein (PRELP), Thrombospondin-4 (THBS4), Lumican (LUM), and multiple collagen isoforms including Collagen Type IV, Collagen Type VI, Collagen Type XII, and Collagen Type XIV [30]. A subset of proteins—Chromodomain-Helicase-DNA-Binding Protein 8 (CHD8), Myosin Light Chain 6B (MYL6B), Filamin-A (FLNA), Liprin-Alpha-1 (PPFIA1), Alpha/Gamma/Beta Enolases (ENO1/ENO2/ENO3), Myosin Heavy Chain 16 (MYH16), and Ribosomal Protein L7-Like 1 (RPL7L1)—was shared between SF and disc tissue, indicating intracellular processes linked to metabolic regulation, cytoskeletal maintenance, and cellular adaptation. Differentially expressed proteins such as PRDX2, TF, and TGFBI were associated with ferroptosis, hypoxia-related stress, and proteoglycan homeostasis, reflecting intracellular mechanisms that remain undetected by classical cytokine-based profiling [14,15].

Proteomic pathway signatures, therefore, allow discrimination between inflammatory, mechanical, and degenerative TMD phenotypes, providing higher diagnostic resolution than isolated biomarkers. Collectively, SF proteomics offers a multidimensional framework for understanding TMD phenotypes through pathway-level organization, supporting future molecular classification and multi-marker diagnostic panel development.

#### 4.4.2. Other “Omics” Approaches (Metabolomics, Matrix Interaction, Angiogenesis and Metabolic Mediators)

Metabolomic studies have expanded the understanding of TMD by identifying molecular alterations that lie beyond the scope of classical inflammatory biomarkers [31]. Recent SF profiling revealed more than 300 metabolites associated with TMJ OA, highlighting disruptions in choline metabolism, arachidonic-acid-derived lipids, and selenocompound biosynthesis, all of which correlate with structural degeneration and symptom severity [31]. These findings suggest that energy handling, membrane turnover, and oxidative lipid processing play key roles in disease progression. Multi-omics integration further shows that metabolic, angiogenic, redox, and mechanotransductive pathways form coordinated molecular networks rather than acting independently. Such interconnected clusters reveal how microvascular remodeling, ECM dynamics, and intracellular stress-response circuits jointly shape TMD phenotypes [15]. Tissue–fluid interaction analyses demonstrate that SF captures only part of the intra-articular molecular landscape: concordance between synovial tissue and fluid is limited for most mediators, with IL-8 being the only consistently overlapping signal [12]. This highlights the need for integrated sampling strategies to fully characterize joint biology. Together, these metabolomic and multi-omics approaches offer a systems-level understanding of TMD, revealing phenotype-specific molecular circuits that extend beyond traditional biomarkers and providing a foundation for future precision-diagnostic frameworks.

### 4.5. Limitations and Barriers to Clinical Translation

Several important limitations must be considered when interpreting the findings of this narrative review. First, the literature search was restricted to PubMed and was performed up to March 2025; studies indexed exclusively in Embase, Web of Science, or Scopus may therefore have been omitted. Second, the included studies are predominantly cross-sectional, with small sample sizes—often fewer than 30 patients per phenotype—and lack independent external validation cohorts, limiting the generalizability of reported findings. Third, the absence of standardized SF collection protocols (variable irrigation volumes, differing arthrocentesis techniques, inconsistent dilution-factor correction) introduces substantial analytical heterogeneity that prevents direct comparability across studies. Fourth, no study in this review has prospectively validated a multi-analyte SF panel for clinical diagnosis, disease staging, or treatment selection; claims about “precision medicine” in this context therefore remain aspirational rather than evidence-based. Fifth, most identified biomarkers—including IL-6, TNF-α, MMP-9, and VEGF—are not specific to TMD and are equally elevated in rheumatoid arthritis, knee osteoarthritis, and other inflammatory arthropathies, raising important questions about diagnostic specificity and clinical utility. Sixth, advanced proteomic workflows such as LC–MS/MS and metabolomics, while analytically powerful, remain inaccessible in routine clinical settings due to high cost, complex sample preparation, and specialized bioinformatic requirements. Multiplex bead-based platforms (e.g., Luminex) may represent a more clinically feasible intermediate step for translating multi-analyte profiling into practice. Future research should prioritize prospective multicenter cohort studies with standardized SF collection protocols—ideally incorporating dilution-factor correction—and test whether baseline SF molecular profiles predict response to specific therapeutic interventions such as arthrocentesis, intra-articular hyaluronic acid injection, or surgical management. Until such evidence emerges, SF biomarker-based precision medicine in TMDs remains a promising but unvalidated paradigm.

## 5. Conclusions

Temporomandibular disorders are characterized by heterogeneous clinical presentations that reflect complex, overlapping biological processes within the temporomandibular joint. Synovial fluid analysis offers a direct window into these mechanisms by capturing molecular alterations associated with inflammation, extracellular matrix degradation, oxidative stress, lubrication failure, and angiogenic activation. The integration of targeted biomarker studies with emerging proteomic and metabolomic approaches has revealed that specific TMD phenotypes exhibit distinct molecular signatures, supporting the concept that these conditions are not merely mechanical disorders but biologically stratified disease entities.

Synovial fluid proteomics, in particular, provides a powerful platform for identifying clinically relevant protein clusters and pathways that may serve as diagnostic, prognostic, or therapeutic markers. Although current evidence highlights strong potential for translation into clinical practice, further progress requires methodological standardization, multicenter validation, and development of clinically applicable analytical workflows.

Analytical heterogeneity across proteomic studies may introduce bias and compromise the comparability of conclusions. A standardized method to collect and analyze SF is strongly recommended to validate further studies. Overall, synovial fluid-based molecular profiling represents a promising avenue toward precision medicine in TMD, with the potential to facilitate earlier diagnosis, improved phenotypic classification, and the future development of individualized therapeutic strategies. However, methodological standardization, prospective validation, and evidence of clinical utility are required before SF biomarker panels can be implemented in routine practice.

## Figures and Tables

**Table 1:** Summary of included studies: study design, patient population, analytical method, main biomarkers investigated, and key findings. DDwR: disc displacement with reduction; DDwoR: disc displacement without reduction; OA: osteoarthritis; RA: rheumatoid arthritis; TMD: temporomandibular disorders; TMJ: temporomandibular joint; ELISA: enzyme-linked immunosorbent assay; LC–MS/MS: liquid chromatography–tandem mass spectrometry; GAG: glycosaminoglycans; ID: internal derangement; NR: not reported; RCT: randomized controlled trial.

Author, Year	Study Design	TMD Phenotype/Population	N	Analytical Method	Main Biomarkers	Key Findings
Bronstein, 1989 [18]	Technical review	Mixed TMD	NR	Arthroscopy/macroscopic SF	SF macroscopic characteristics	First description of arthroscopic TMJ SF collection; macroscopic differences between healthy and diseased joints noted
Aghabeigi et al., 1993 [19]	Case series	Internal derangement; RA	2	Biochemical analysis	Protein composition, inflammatory mediators	SF protein profiles differ in inflammatory TMD; SF analysis proposed as clinically useful tool
Zardeneta et al., 1997 [20]	Prospective	Internal derangement; OA	NR	ELISA, protein assay	Total protein, albumin, fibronectin	Protein concentration decreases proportionally with irrigation volume; standardised collection protocol essential
Murakami et al., 1998 [21]	Cross-sectional	Internal derangement	NR	HPLC, spectrophotometry	PGE2, hyaluronic acid, chondroitin-4 and -6 sulfates	PGE2 elevated in internal derangement; chondroitin sulfates reflect fibrocartilage breakdown
Shibata et al., 1998 [22]	Cross-sectional	DDwR, DDwoR, OA, RA vs. healthy	30 joints	HPLC with fluorometry	GAG components (chondroitin-4-S, chondroitin-6-S, HA)	GAG profiles differ by phenotype; chondroitin sulfate composition correlates with degenerative stage
Alstergren et al., 1999 [23]	Methodological	Mixed TMD	NR	Spectrophotometry (hydroxocobalamin)	Dilution factor, true SF volume	Hydroxocobalamin method enables accurate SF volume quantification; dilution correction mandatory
Takahashi et al., 1999 [24]	Cross-sectional	DDwR, DDwoR, OA vs. healthy	75 joints	Griess reaction (NO metabolites)	Nitric oxide (NO, nitrite)	NO significantly elevated in DDwoR and OA; correlates with degenerative severity
Tobe et al., 2002 [25]	In vitro	TMJ synovial cell cultures	NR	ELISA, PCR	IL-1beta, IL-8	IL-1beta stimulates IL-8 in TMJ synovial cells; supports cytokine cascade in synovitis
Kaneyama et al., 2002 [26]	Cross-sectional	Internal derangement, OA	121 joints	ELISA	IL-1beta, TNF-alpha, IL-6, IL-8	All cytokines elevated across TMD groups; IL-1beta and IL-6 highest in OA
Alstergren et al., 2003 [13]	Cross-sectional	Chronic polyarthritides with TMJ involvement	NR	ELISA	IL-1beta, IL-1Ra, sIL-1RII	IL-1Ra present in all SF; IL-1 system dysregulation confirmed in polyarthritis-related TMD
Gulen et al., 2009 [10]	Prospective interventional	Internal derangement, OA	NR	ELISA	IL-1beta, IL-6, TNF-alpha	Cytokines significantly reduced post-arthrocentesis; SF lavage exerts anti-inflammatory effect
Herr et al., 2011 [17]	Pilot case–control	TMD with clicking vs. controls	6 subjects	iTRAQ-MS, protein arrays	EG-VEGF/PK1, D6, multiple proteins	Proof-of-concept proteomics; candidate proteins differentiate TMDs from healthy controls
Kim et al., 2012 [27]	Cross-sectional	Normal TMJ (orthognathic controls) vs. TMD	34 patients	ELISA	IL-1beta, IL-2, IL-4, IL-5, IL-6, IL-8, IL-10, TNF-alpha, IFN-gamma	Normative cytokine baseline for healthy TMJ SF established
Kellesarian et al., 2016 [11]	Systematic review	All TMD phenotypes (pooled)	Multiple studies	ELISA (primary studies)	IL-1beta, IL-6, IL-8, TNF-alpha, IFN-gamma	IL-1beta, IL-6, TNF-alpha reproducibly elevated; IL-6 most consistently linked to OA
Kristensen et al., 2014 [5]	Cross-sectional	Healthy TMJ (JIA controls)	NR	ELISA, multiplex	IL-1beta, IL-6, IL-8, IL-10, TNF-alpha, IFN-gamma	Reference cytokine values for healthy TMJ SF established; essential normative dataset
Ozdamar et al., 2017 [28]	RCT	DDwR/DDwoR (symptomatic ID)	24 patients	Spectrophotometry	Myeloperoxidase (MPO)	MPO elevated pre-treatment; HA injection reduces MPO more than saline alone
Loreto et al., 2020 [29]	Cross-sectional	Severe TMJ dysfunction (synovial tissue)	NR	Immunohistochemistry	MMP-7, MMP-9	MMP-7 and MMP-9 overexpressed in severe TMD synovial tissue; fibrocartilage remodelling confirmed
Doetzer et al., 2021 [14]	Cross-sectional	DDwoR, condylar hyperplasia, dislocation	NR (pilot)	nanoLC–MS/MS	HSPB1, SERPINF1, THBS3, TNC, LGALS1, LUM, PKM, S100A10, SERPINA1, SOD3, TF, TGFBI	First nanoLC–MS/MS TMJ SF study; phenotype-specific clusters; ferroptosis and hypoxia pathways identified
Ulmner et al., 2021 [30]	Cross-sectional	Mixed TMD (surgical candidates)	NR	Proteomics, ELISA	Synovial tissue proteins, clinical variables	Combined SF/tissue protein profiles predict surgical outcomes
Ulmner et al., 2022 [12]	Cross-sectional	DDwR, DDwoR, OA	NR	ELISA, multiplex	IL-1beta, IL-6, IL-8, IL-10, TNF-alpha (SF and tissue)	Poor SF-tissue cytokine correlation (except IL-8); SF alone underestimates intra-articular inflammation
Liu et al., 2022 [15]	Cross-sectional	DDwR, DDwoR vs. healthy controls	NR	LC–MS/MS proteomics	Collagen isoforms (IV, VI, XII, XIV), PRDX1/2, PRELP, THBS4, LUM, ECM proteins	Phenotype-specific proteomic clusters; shared SF-disc proteins linked to metabolic regulation
Zhang et al., 2024 [31]	Cross-sectional	TMJOA (mild, moderate, severe)	90 patients	Untargeted metabolomics (LC–MS)	Choline metabolites, arachidonic acid derivatives, selenocompounds (>1498 metabolites)	Metabolomics stratifies TMJOA by grade; 9 diagnostic biomarkers identified
Maffia et al., 2026 [32]	Pilot technical study	Mixed TMD	NR (pilot)	Closed-loop dual-needle arthrocentesis	Total protein yield, SF volume	Closed-loop circuit reduces dilution; improves protein yield for downstream analysis

## Data Availability

No new data were created or analyzed in this study. Data sharing is not applicable to this article.
